# hTERT associates with Rab7A to enhance autophagy and rescue oxLDL induced endothelial dysfunction

**DOI:** 10.3389/fcvm.2026.1882233

**Published:** 2026-09-25

**Authors:** Praveen Machiraju, Rajesh Srinivas, Rohit Shetty, Rupak Mukhopadhyay, Stephane Heymans, Arkasubhra Ghosh

**Affiliations:** 1 GROW Research Laboratory, Narayana Nethralaya Foundation, Bangalore, India; 2 Department of Cardiology, Cardiovascular Research Institute Maastricht (CARIM), Maastricht University, Maastricht, Netherlands; 3 Department of Vascular and Endovascular Surgery, Narayana Health, Bangalore, India; 4 Department of Cornea and Refractive Surgery, Narayana Nethralaya, Bangalore, India; 5 Faculty of Health, Medicine and Life Science, School Mental Health and Neurosciences, Maastricht University, Maastricht, Netherlands; 6 Department of Molecular Biology and Biotechnology, Tezpur University, Napaam, India; 7 Department of Cardiovascular Sciences, Centre for Molecular and Vascular Biology, KU Leuven, Leuven, Belgium

**Keywords:** atherosclerosis, autophagy, endothelial dysfunction, RAB7A, telomerase reverse transcriptase

## Abstract

**Introduction:**

Endothelial dysfunction is a hallmark of atherosclerosis, driven by chronic exposure to oxidized LDL and linked to impaired autophagy. Although restoring autophagy in atherosclerosis has been shown to yield protective effects, the mechanisms contributing to dysregulated autophagy are unknown. Apart from previously known extra telomeric role in regulating inflammation, emerging evidence suggests a strong direct correlation between hTERT and autophagy pathway, however, potential molecular mechanisms still remain unknown. This study investigates hTERT's extra-telomeric function in rescuing oxLDL-induced endothelial dysfunction via autophagy regulation.

**Methods:**

hTERT was ectopically overexpressed or knocked down in oxLDL treated HUVEC cells to study its effect in endothelial dysfunction using oxLDL retention, monocyte attachment, endothelial resistance and barrier breach assays. Autophagy-related gene expression and autophagy dynamics were evaluated through qPCR, western blotting, live-cell imaging, immunofluorescence, and co-immunoprecipitation.

**Results:**

OxLDL-induced endothelial dysfunction reduced hTERT expression and increased ICAM1 levels. Pharmacological inhibition of hTERT with MST312 upregulated ICAM1 indicating induction of endothelial dysfunction. Ectopic hTERT expression rescued endothelial dysfunction, demonstrated by reduced intracellular oxLDL levels, decreased monocyte adhesion, improved barrier integrity, and increased TEER. Conversely, hTERT knockdown exacerbated these dysfunction markers. Mechanistically, hTERT enhanced autophagy gene expression and kinetics, with hTERT blockade reducing lysosome abundance. hTERT colocalized and associated with Rab7A under basal conditions, with enhanced association following oxLDL exposure.

**Conclusion:**

This study highlights a novel additional role of extra telomeric hTERT in contributing to endothelial resilience against atherogenic stress by rescuing endothelial dysfunction via enhancing the expression of autophagy-related gene expression and enhancing autophagy dynamics by associating with Rab7A.

## Graphical Abstract

**Figure:**
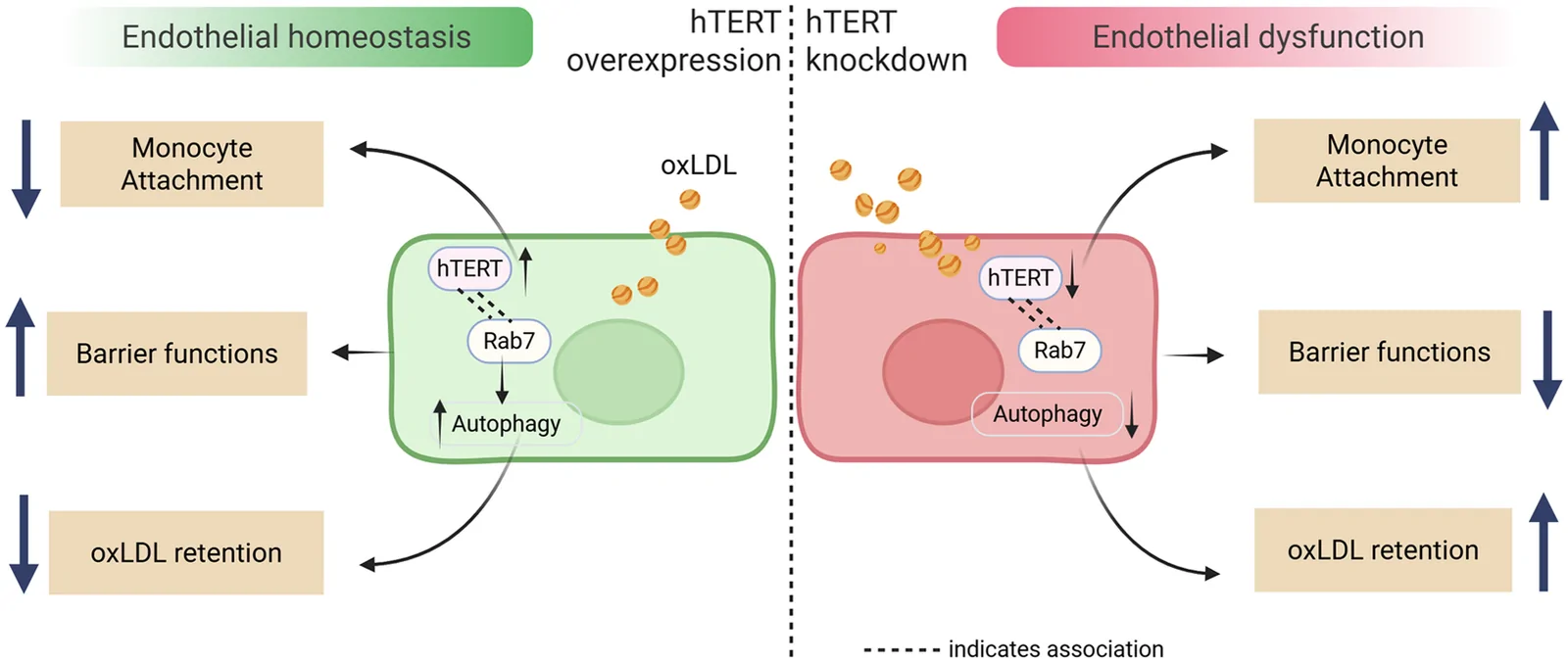
hTERT protects against endothelial dysfunction by associating with Rab7A and positively regulating autophagy (Created using BioRender.com).

## 1 Introduction

Atherosclerosis remains a leading cause of morbidity and mortality worldwide, with a global prevalence of approximately 5.6% among adults aged 25 years and older (1). At the core of atherogenesis is endothelial dysfunction, a pathological state characterized by increased expression of adhesion molecules such as intercellular adhesion molecule-1 (ICAM1) and vascular cell adhesion molecule-1 (VCAM1) and loss of barrier integrity (2–4). These functional impairments facilitate subendothelial accumulation of modified lipoproteins and triglyceride-rich particles (5, 6). Concomitantly, the upregulation of adhesion molecules promotes leukocyte adhesion and transmigration, fueling a cycle of local inflammation and plaque development (7, 8).

Autophagy is a key pathway that is dysregulated in atherosclerosis, a critical housekeeping mechanism responsible for degradation and recycling in the cells. In endothelial cells, impaired autophagy leads to accumulation of reactive oxygen species (ROS), dysregulation of multiple associated pathways and mitochondrial dysfunction, setting the stage for plaque formation. Recent studies have demonstrated that endothelial Rab7 deficiency causes dysregulation of autophagy, mitochondrial dysfunction, and endothelial senescence, directly contributing to vascular pathology (9–12). Remarkably, restoring autophagy ameliorated atherogenic phenotypes (13–15). Since endothelial dysfunction marks the beginning of vascular pathologies which are often irreversible, it is imperative to understand mechanisms leading to endothelial dysfunction.

Long-term exposure to atherogenic insults, particularly oxidized LDL (oxLDL), has been shown to induce a senescence-like phenotype in endothelial cells, suggesting that molecular aging contributes to endothelial dysfunction and atherogenesis (16, 17). A molecular hallmark of senescence is downregulation of the catalytic subunit of the human telomerase reverse transcriptase (hTERT) (18). Beyond telomere maintenance (19), hTERT also exerts diverse non-canonical functions, including modulation of inflammatory signaling (20), Wnt/*β*-catenin pathway regulation (21), miRNA expression (22), and cellular responses to DNA damage (23, 24). Clinically, dysregulation of hTERT has been implicated in degenerative conditions such as idiopathic pulmonary fibrosis and aplastic anemia (25–27). And more recently, hTERT has been shown to participate in autophagy regulation in other systems (28, 29).

Despite these findings, mechanistic insights at cellular level as well as the functional relevance of hTERT and its related mechanisms in modulating autophagy particularly under pro-atherogenic conditions, remains poorly understood. Recently published transcriptomic analyses of human atherosclerotic plaques has revealed novel associations between telomerase-related gene expression and autophagy networks (30), suggesting an unexplored regulator*y* axis. Given that both autophagy impairment and hTERT downregulation are observed in atherosclerosis, we hypothesized that reduction of endothelial hTERT levels due to exposure of oxLDL leads to autophagy dysfunction, precipitating the pathological features in atherosclerotic vessels. The study, thus aims to dissect out a novel hTERT autophagy regulator*y* axis in endothelial dysfunction in context of atherosclerosis.

## 2 Materials and methods

### 2.1 Cell culture and treatments

Human umbilical vein endothelial cells (HUVEC, Cat.no.C2519A, Lonza) were cultured in complete endothelial growth medium at 37 °C with 5% CO₂. Cells were treated with 40 μg/mL oxidized LDL for 96 h prior to gene and protein expression analysis. For oxLDL imaging, cells were incubated with 20 μg/mL DiI-labelled oxLDL (DiI-oxLDL, Cat.no.L34358, Invitrogen) for 1 h. Autophagy dynamics using western blotting was assessed by pre-treating cells with either trehalose (100 mM, autophagy inducer, Cat.no.103097, MP Biomedicals) or chloroquine (5 μM, lysosomal inhibitor, Cat.no.C6628, Sigma) for 24 h before harvest. hTERT catalytic activity was blocked using 5 μM MST312 (Cat.no.M3949-25MG, Sigma). hTERT activity was verified 96 h post-treatment using real-time PCR-based telomerase repeat amplification protocol (TRAP) assay as previously described (31).

### 2.2 Transient transfections

HUVEC cells were transfected using lipofectamine LTX Plus reagent (Cat.no.15338100, Invitrogen). 2 µg of plasmid DNA – pWZL-hTERT for overexpression (A kind gift from William Hahn, Addgene plasmid # 22396) transEDIT ™ pCLIP-gRNA-hTERT for hTERT knockdown along with respective vector controls were used for transfection. Briefly, 2 µL of PLUS reagent, mixed with 2 µg of plasmid and 2 µL of lipofectamine were incubated at room temperature separately for 5 min, plasmid containing mixture was added dropwise to lipofectamine and incubated for 15 min at room temperature (RT). The transfection mix was then added dropwise to HUVEC cells in a 6-well dish and incubated for 16 h before complete media change. Three single guide RNAs (sgRNAs) were tested at both mRNA and protein expression levels, wherein sgRNA3 was observed to be most efficient in reducing hTERT levels. We further tested two top performing sgRNAs (sgRNA1 and sgRNA3) for functional validation and sgRNA3 demonstrated the most potent functional outcomes. It was therefore used for all further experiments (Supplementary Figure S1). Rab7A based experiments were performed using Rab7DN plasmid [A kind gift from Richard Pagano (Addgene plasmid # 12660)] by transfecting 500 ng of plasmid into cells in a 12 well plate. Autophagy kinetics were studied using LC3 dual reporter plasmid, by transfecting pCDH-CMV-mC-G-LC3B-P plasmid (A kind gift from Kazuhiro Oka, Addgene plasmid # 124974) into HUVEC cells. Plasmid concentrations and Lipofectamine reagent ratios were adjusted according to plate format.

### 2.3 RNA isolation and real time PCR

Total RNA was isolated from HUVEC cells using TRIZOL® method according to manufacturer's protocol (Cat.no.15596018, Invitrogen). 1,000 ng RNA was converted into cDNA using Bio-Rad iSCRIPT cDNA conversion kit (Bio-Rad, Philadelphia, PA, USA). Real time PCR was performed using SYBR green reagent (Cat.no.KK4602, Roche). The quantitative real-time PCR cycle included denaturation at 95 °C for 3 min, 40 amplification cycles at 95 °C for 10 s, 58 °C for 30 s using a CFX Connect TM real-time PCR detection system (Bio-Rad, Philadelphia, PA, USA). Expression levels were determined by normalizing the expression of these genes to *β*-actin in the respective samples. Supplementary Table S1 lists out primer sequences used in the study.

### 2.4 Western blotting

Cells were harvested by scraping in ice-cold PBS. Protein fraction was isolated by incubating the cell pellet in radioimmunoprecipitation assay (RIPA) buffer for 30 min with constant agitation followed by centrifugation at 13,000 rpm at 4 °C for 15 min. Proteins were quantified using Bradford assay and 20 µg of total protein was used for separation on SDS-PAGE followed by western blotting. Western blotting was performed by transferring proteins to PVDF membrane and incubating with primary antibodies at a dilution of 1:1,000 overnight at 4 °C. Secondary antibodies were used at a dilution of 1:5,000 and incubated for 1 h at RT. The blots were developed using ECL (Cat.no.1705060, Bio-Rad). Supplementary Table S2 lists out details of antibodies used in the study.

### 2.5 LDL oxidation and Oil Red O staining

LDL (Cat.no.LP2-2MG, Sigma) was oxidized using copper sulphate pentahydrate (CuSO_4_.5H_2_O, Cat.no.61298, SRL). 1 mg of LDL was incubated in 1 mL PBS containing 10 µM CuSO_4_.5H_2_O for 24 h at 37 °C in a 35 mm dish. Oxidation of LDL was monitored using absorption at 234 nm as previously described (32). After the OD crossed 1.00 at 234 nm, the reaction was stopped by adding 5 mM EDTA followed by filtration using 100 kDa cut off amicon filters to remove free copper. The oxidized LDL was resuspended in 1 mL of PBS and stored at 4 ^o^C and used within 1 week of preparation for *in vitro* experiments. Protein concentration was assessed using Bradford assay and Oil Red O staining was performed to visualize lipids in cells. 4% paraformaldehyde fixed cells were incubated with Oil Red O stain working solution for 30 min at RT followed by washing with dH2O and 60% isopropanol to remove excess stain. The cells were then imaged using brightfield microscopy (Olympus CKX53). Oil Red O staining was quantified by washing the cells with 100% isopropanol and measuring the absorbance at 495 nm.

### 2.6 Immunofluorescence and live cell imaging

Cells were fixed using 4% paraformaldehyde for 10 min at room temperature (RT), followed by washing with phosphate buffered saline (PBS). Blocking was performed using 3% bovine serum albumin (BSA) in Tris-buffered saline with 0.1% Tween-20 (TBST) containing 100 mM of glycine. Primary antibodies were used at a dilution of 1:500 and incubated overnight at 4 °C followed by secondary antibody incubation at a dilution of 1:3,000 for 2 h at RT. Cells were then washed with PBS and mounted using Fluoroshield containing DAPI (Cat.no.F5067, Sigma). For live imaging of lysosomes, untreated and MST312 treated HUVEC cells were incubated with lysotracker red stain (Cat.no.L7528, Thermo Scientific) for at a dilution of 1:10,000 30 min. The unbound stain was washed off using PBS and the cells were imaged using Nanolive ™ 3D cell explorer-fluo (Nanolive SA, Switzerland). Live cell imaging was performed for 18 h at time intervals of 5 min/frame. Barrier integrity was studied using ZO-1 staining where ZO-1 was used at a dilution of 1:200 and secondary Cy3 antibody was used at a dilution of 1:5,000.

### 2.7 Monocyte attachment assay

Monocyte attachment assay was performed using THP-1 monocyte cells stained with calcein. Calcein staining was performed for 30 min using 10 µM calcein for 10 × 10^6^ cells. THP-1 cells were washed with PBS to remove the excess stain. 2 × 10^6^ calcein stained cells were incubated with hTERT overexpressed and hTERT knocked down HUVEC cells for 30 min. Unbound THP-1 cells were washed off using PBS and co-cultured cells were imaged using EVOS FL microscope.

### 2.8 Trans endothelial/epithelial resistance assay (TEER) and barrier breach assay

TEER assay was performed by measuring resistance across monolayer of HUVEC cell cultured on a transwell plate using a voltohm meter (Millicel ERS-2, Millipore, MERS0002) according to manufacturer's protocol. To assess barriers breach, 40 kDa FITC dextran (Cat.no.FD40, Sigma) was incubated for 30 min on HUVEC cells cultured on a transwell plate. Fluorescence readings were obtained using a multiwell plate reader (TECAN Spark, Switzerland) with 488 nm excitation and 550 nm emission filters for both the bottom chamber media and top chamber media.

### 2.9 Relative telomeric length assay (RTL)

RTL was performed using quantitative real time PCR as described previously (33). Two primers against Telomere repeats and a single copy gene, human beta globin were used to quantify telomeric repeats relative to single copy gene.

### 2.10 Co-immunoprecipitation

5 × 10^6^ HUVEC cells were used for Co-immunoprecipitation (Co-IP) experiments. The cells were harvested in IP lysis buffer and 1 mg of total protein was incubated with 2 µL of hTERT antibody overnight at 4 °C. Protein A/G beads (Cat.no.17528004,17061802, GE Healthcare) were pre blocked using 3% BSA. Lysate and antibody were incubated overnight at 4 °C followed by incubation with beads for 4 h at 4 °C. The beads were then spun down at 1,000 rpm for 1 min followed by six washes with IP wash buffer and lysed using 6× laemmli buffer. Laemmli buffer containing denatured pulled down proteins were used for western blotting. 1% input was used for all immune precipitation experiments.

### 2.11 Statistical analysis

For patient sample gene expression analyses, paired t-test was used. For quantification of immunofluorescence images, mean fluorescence intensities (MFI) were measured for a minimum of two fields per image using Fiji software. All the gene expression studies were performed in two technical replicates. Three independent repeats were used for statistical analysis of mean fluorescence intensities as well as gene and protein expression. Mean fluorescent intensities of lysotracker stain and DiI-oxLDL were normalized to DAPI intensity to account for variability in cell numbers. All *in-vitro* experiment data sets were analyzed using unpaired two tailed test to test the significance. All data in graphs are presented as Mean ± SEM. For analysis of imaging-based autophagy flux assay using LC3 dual reporter plasmid, cells positive for green fluorescent protein (GFP), red fluorescent protein (RFP) and DAPI were counted for yellow and red puncta using Fiji software. Two fields per condition and a minimum of 5 images per field were counted. The mean of yellow and red puncta for all three biological replicates was converted to percentage values and represented in the graph.

## 3 Results

### 3.1 Autophagy is dysregulated in peripheral artery disease

From our previously published transcriptomic study in PAD patients (30) where atherosclerotic plaque tissues were compared to non-atherosclerotic control regions from same patients (*n* = 9), we analyzed the expression levels of autophagy related genes. Our analyses revealed significant dysregulation of autophagy-related genes, including ATG3 (log2FC = −0.2, *p* ≤ 0.005), RAB7A (log2FC = −0.15, *p* ≤ 0.005), RAB11 (log2FC = −0.51, *p* ≤ 0.05), and BECN1 (log2FC = −1.8, *p* ≤ 0.05), as well as autophagy regulatory genes USP8 (log2FC = −0.83, *p* ≤ 0.005) and UBQLN1(log2FC = −0.42, *p* ≤ 0.05) (Figures 1A,B). Overall, autophagy pathway was significantly altered in atherosclerotic tissues.

**Figure 1:**
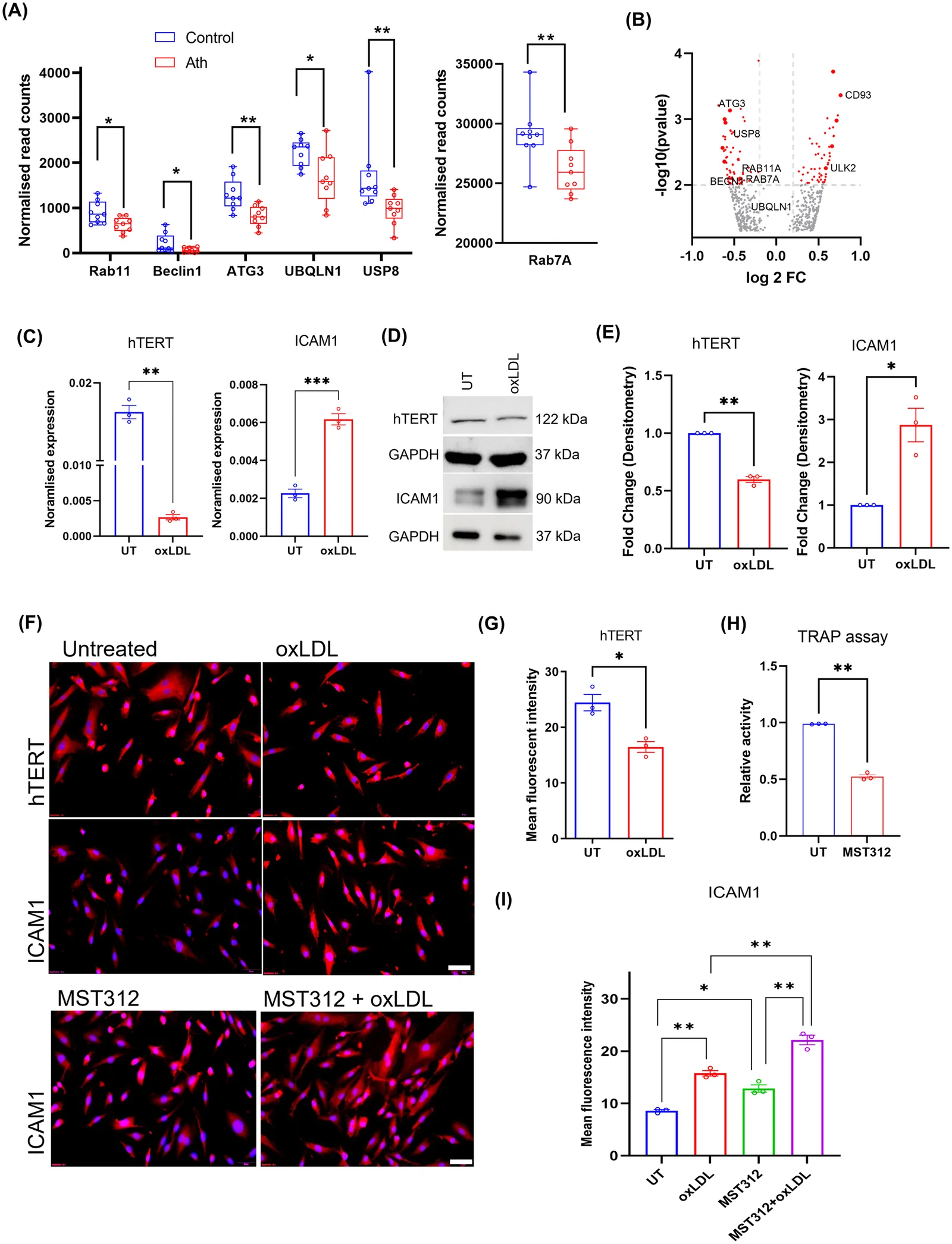
oxLDL reduces hTERT expression and induces endothelial dysfunction. **(A)** Normalized read counts of autophagy related genes obtained from peripheral artery disease patient samples compared to paired controls (*n* = 9) (paired t-test, *p < 0.05, **p < 0.0005). **(B)** Volcano plot showing autophagy related genes obtained from previously published transcriptomic study **(C)** Gene expression analysis of hTERT and ICAM1 genes in HUVEC cells under 40 µg/mL oxLDL treatment for 96 h. **(D)** Western blot of hTERT and ICAM1 proteins in HUVEC cells upon oxLDL treatment. **(E)** Densitometric analysis of protein expression levels. **(F)** Immunofluorescent images of hTERT and ICAM1 under oxLDL and 5 µM MST312 in HUVEC cells. Scale bar =50 µm **(G and I)** Mean fluorescence intensity quantifications of immunofluorescence images. **(H)** TRAP assay to determine hTERT activity blockage upon MST treatment. All experiments are representative on *n* = 3 biological replicates. Data are presented as Mean ± SEM, Unpaired two-tailed t-test, *p < 0.05, **p < 0.005, ***p < 0.0005.

### 3.2 oxLDL reduces hTERT expression

HUVEC cells treated with 40 µg/mL of oxLDL for 96 h showed a significant reduction in the gene expression of hTERT (log2FC = −2.6, *p* ≤ 0.005) and concomitant induction of ICAM1 expression (log2FC = 1.44, *p* ≤ 0.005; Figure 1C). Protein expression analysis following oxLDL treatment yielded similar results (Figure 1D). hTERT protein expression levels showed a significant reduction (*p* < 0.005) whereas ICAM1 showed a 3-fold increase in protein expression (*p* < 0.05) (Figure 1E). Immunofluorescence staining following oxLDL treatment also showed significant reduction in hTERT expression (*p* < 0.005; Figures 1F,G). ICAM1 expression significantly increased following treatment. We observed that MST312 treatment of HUVEC cells alone considerably increased ICAM1 expression as compared to untreated cells indicating that hTERT inhibition contributed directly to this endothelial dysfunction like phenotype (Figure 1I). 5 µM MST312 treatment for 24 h reduced hTERT activity by 40% (*p* < 0.005) (Figure 1H).

### 3.3 hTERT protects against endothelial dysfunction

Following our observation that hTERT inhibition induced a dysfunctional phenotype, we assessed the functional outcomes of hTERT overexpression (hTERT Oe) and knockdown (hTERT Kd) in HUVEC cells. Protein and gene expression analysis through western blotting and real time PCR showed significant upregulation and knockdown of hTERT upon transfection (Supplementary Figure S2A–C). Functional assays revealed the protective role of hTERT upon exposure to oxLDL. Overexpression of hTERT significantly reduced the intracellular oxLDL levels (FC = 0.5, *p* < 0.0005) whereas hTERT Kd significantly increased oxLDL retention as assessed by DiI labelled oxLDL imaging (FC = 2.45, *p* < 0.0005; Figures 2A,B; Supplementary Figure S2C). hTERT Oe also reduced the total lipid content inside the cells and hTERT Kd increased total lipid content as assessed by Oil Red O staining (Supplementary Figure S3A,B). Ectopic hTERT Oe and hTERT Kd transfections did not lead to loss in cell viability even in the presence of oxLDL at the doses used (Supplementary Figure S3C). Further, there was no significant difference in relative telomeric lengths in these cells as observed in RTL assay (Supplementary Figure S3D), indicating that these observations may represent a non-canonical function of hTERT. The RTL is reduced after several rounds of passaging (34). Monocyte attachment assay using calcein stained THP-1 cells showed a significant increase in the attachment of monocytes to HUVEC cells upon oxLDL treatment (FC = 34.4, *p* < 0.005). hTERT Oe reduced monocyte attachment in oxLDL treated HUVEC cells (FC = 0.4, *p* < 0.005). HUVEC cells with hTERT Kd upon oxLDL treatment showed significantly higher monocyte attachment as compared to control and oxLDL treated HUVEC cells (FC = 7.8, *p* < 0.005; Figures 2C,D). We also observed that hTERT Kd HUVEC cells showed a much higher attachment of THP-1 cells as compared to hTERT Oe HUVEC cells indicating that hTERT Kd cells attracted more monocytes for attachment, suggesting a concomitant increase in adhesion factors. In TEER and barrier breach assays, hTERT Oe cells showed higher resistance (FC = 2.05, *p* < 0.005) and lower barrier breach (FC = 0.15, *p* < 0.00005) as compared to hTERT Kd cells when challenged with oxLDL (Figures 2E,F). We assessed the barrier forming capacity using ZO-1 immunostaining in HUVEC cells cultured on transwell membranes. Endothelial barrier integrity was impaired upon hTERT Kd in presence of oxLDL and hTERT Oe cells resisted barrier disintegration upon oxLDL challenge (Figures 2G,H; Supplementary Figure S4). All functional assays were performed 48 h post transfection. The data together indicates that hTERT overexpression rescued oxLDL induced endothelial dysfunction (Figure 2I). Furthermore, we observed that hTERT knockdown aggravated oxLDL induced endothelial dysfunction.

**Figure 2:**
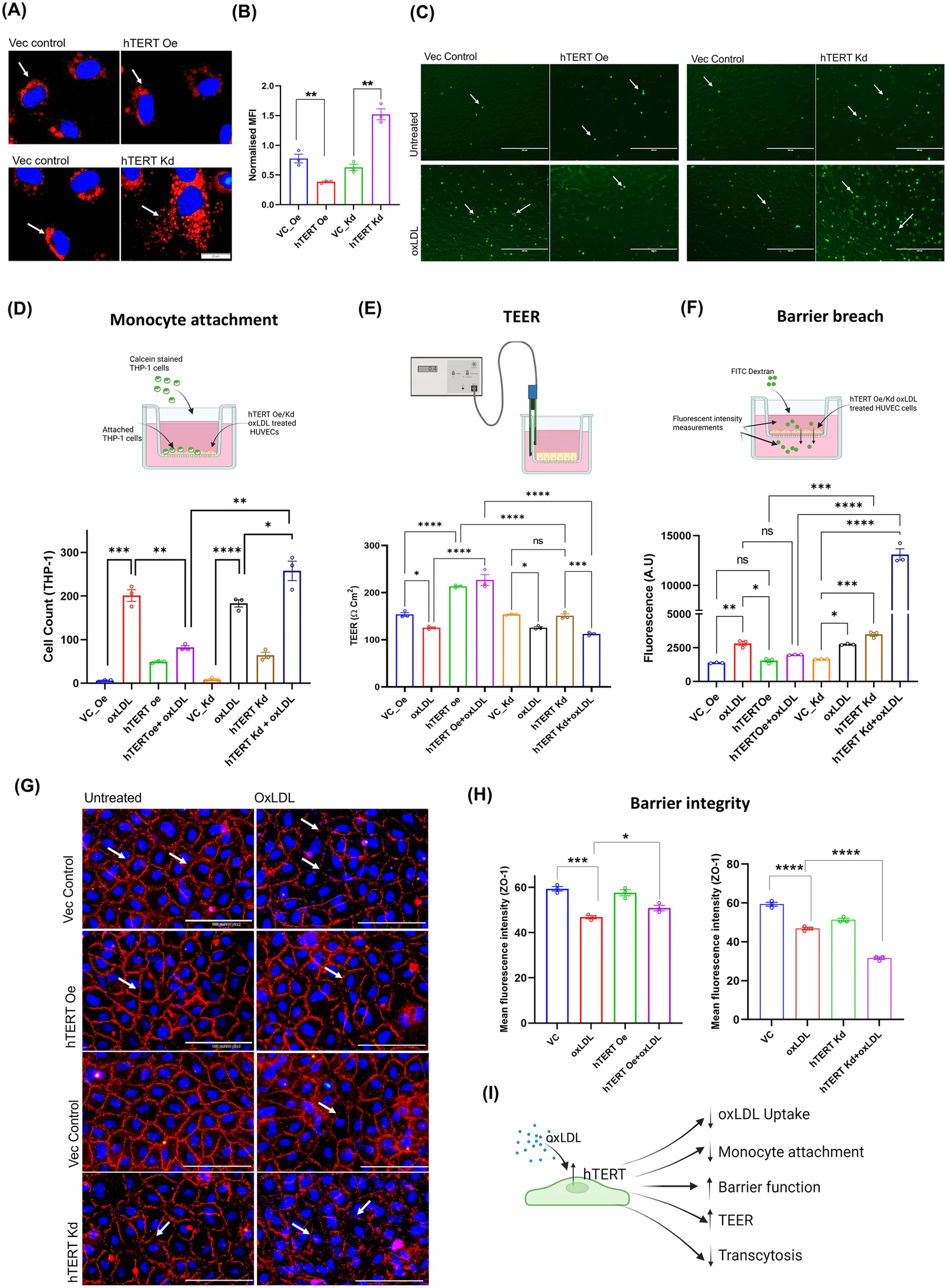
hTERT protects against endothelial dysfunction. **(A)** Enlarged images of DiI-oxLDL in HUVEC cells under hTERT overexpression (hTERT Oe) and hTERT knockdown (hTERT Kd) conditions. Vec Control represents respective vector controls. Corresponding full size raw images are given in Supplementary Figure S2C. **(B)** Quantification of DiI-oxLDL fluorescence intensity under different conditions. **(C)** Calcein stained THP1 monocyte (shown in green by arrows) attached to HUVEC cells transfected with hTERT overexpression and hTERT knockdown plasmids. **(D)** Quantification of cell counts for monocyte attachment assay. **(E)** Trans endothelial resistance (TEER) measured from HUVEC cells grown on transwell plates using voltohm meter. **(F)** Barrier breach quantification performed using HUVEC cells cultured on transwell plates using 40 kDa FITC dextran. **(G)** Enlarged Immunofluorescence images showing HUVEC cells expressing ZO-1, white arrows indicate tight junctions. Corresponding full size raw images are given in Supplementary Figure S4 **(H)** ZO-1 quantification. **(I)** Schema showing rescue effects of hTERT (Created using BioRender.com). All data are representative of *n* = 3 biological replicates. Data are presented as Mean ± SEM. Scale bars =20 µm **(A)**, 400 µm **(C)**, 100 µm **(G)** Unpaired two-tailed t-test, *p < 0.05, **p < 0.005, ***p < 0.0005, ****p < 0.00005.

### 3.4 hTERT regulates expression of autophagy related proteins

Since hTERT deficient HUVEC cells retained more oxLDL as compared to hTERT Oe cells, we speculated that hTERT might help regulate intracellular levels of oxLDL. Therefore, we tested for autophagy related protein and gene expression levels. Overexpression of hTERT significantly increased the levels of autophagy related genes and knockdown of hTERT reduced the levels of autophagy related genes 48 h post transfection (Figure 3A). Protein expression levels of LAMP1 and Rab7A correlated with hTERT expression (Figures 3B,C). To assess the functional outcome of this effect, we analyzed lysosomal content in cells using Lysotracker staining. hTERT Oe cells exhibited significantly higher (FC = 1.56, *p* < 0.00005) lysotracker intensity and hTERT Kd had significantly lesser (FC = 0.43, *p* < 0.0005) lysotracker intensity as compared to respective vector controls (Figures 3D,E; Supplementary Figure S5).

**Figure 3:**
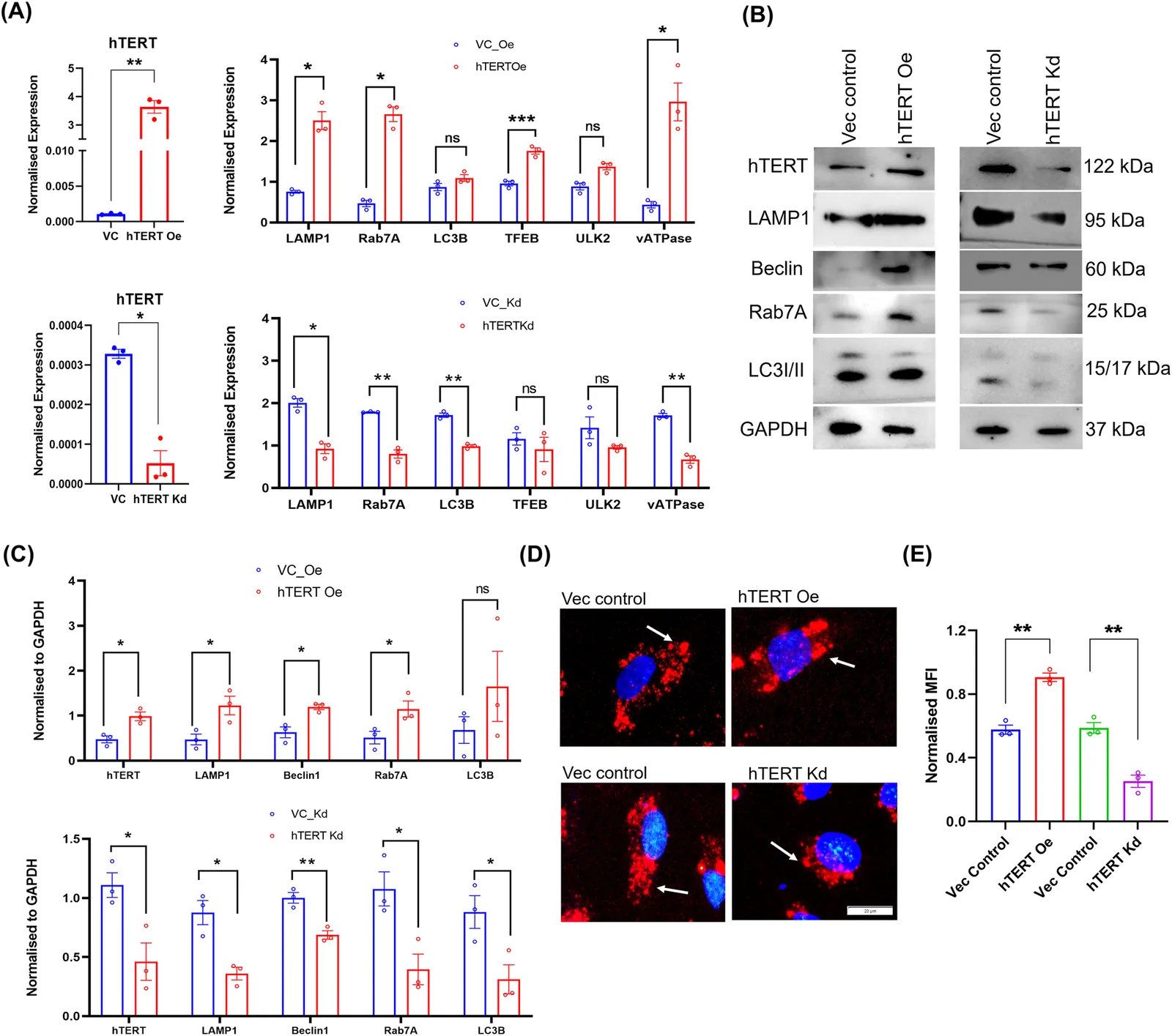
hTERT regulates expression of autophagy related genes and proteins. **(A)** Gene expression analysis of autophagy related genes in HUVEC cells with modulation of hTERT expression. **(B,C)** Western blot showing expression levels of autophagy associated proteins, graph shows densitometric analysis of three independent biological replicates. **(D,E)** Enlarged images of Lysosomes imaged using lysotracker® in HUVEC cells and quantification. Corresponding full size raw images are given in Supplementary Figure S5. White arrows indicate lysosomes. All data are representative of *n* = 3 biological replicates. Scale bar=20 µm. Data are presented as Mean ± SEM, Unpaired two-tailed t-test, *p < 0.05, **p < 0.005, ***p < 0.0005, ****p < 0.00005.

### 3.5 hTERT regulates autophagy dynamics

To test whether hTERT regulates autophagy dynamics apart from regulating the levels of autophagy related genes, we activated autophagy using 100 mM trehalose and inhibited autophagy using 5 µM chloroquine in HUVEC cells either overexpressed or knocked down with hTERT. Trehalose treatment led to accumulation of LAMP1 and induction of Rab7A indicating activation of autophagy in vector controls (Figure 4A, Lane 2). Chloroquine treatment also resulted in an increase in LAMP1 accumulation along with reduction in LC3B and Rab7A (Figure 4A, Lane 3). We observed that overexpression of hTERT enhanced the activation of autophagy when treated with trehalose as evident from increased levels of LAMP1, Rab7A and LC3 (Figure 4A, Lane 4). With chloroquine treatment, hTERT overexpression was associated with higher expression of LAMP1 and Rab7A as compared to vector control; because chloroquine blocks degradation, these steady-state differences reflect altered autophagy-associated protein abundance instead of restored lysosomal degradation (Figure 4A, Lane 6). hTERT Kd did not show rescue of protein expression levels even with trehalose treatment and autophagy inhibition. Using choloquine in hTERT Kd cells further reduced expression levels of autophagy related genes (Figure 4B). TFEB is a key transcriptional factor involved in the transcription of autophagy related genes, we observed a reduction in expression levels of TFEB upon hTERT knockdown, similar effect was seen in expression of v-ATPase, a proton pump protein required for proper functioning of lysosomes (Figures 4C,D) indicating that hTERT knockdown downregulates autophagy associated proteins. Autophagy flux assessment using dual reporter assay showed a significant increase in mature red puncta in hTERT overexpressed HUVEC cells as compared to vector control and significantly increased yellow puncta and reduced red puncta in hTERT knockdown cells showing reduced autophagy flux, consistent with results from western blotting (Figures 4E,G).

**Figure 4:**
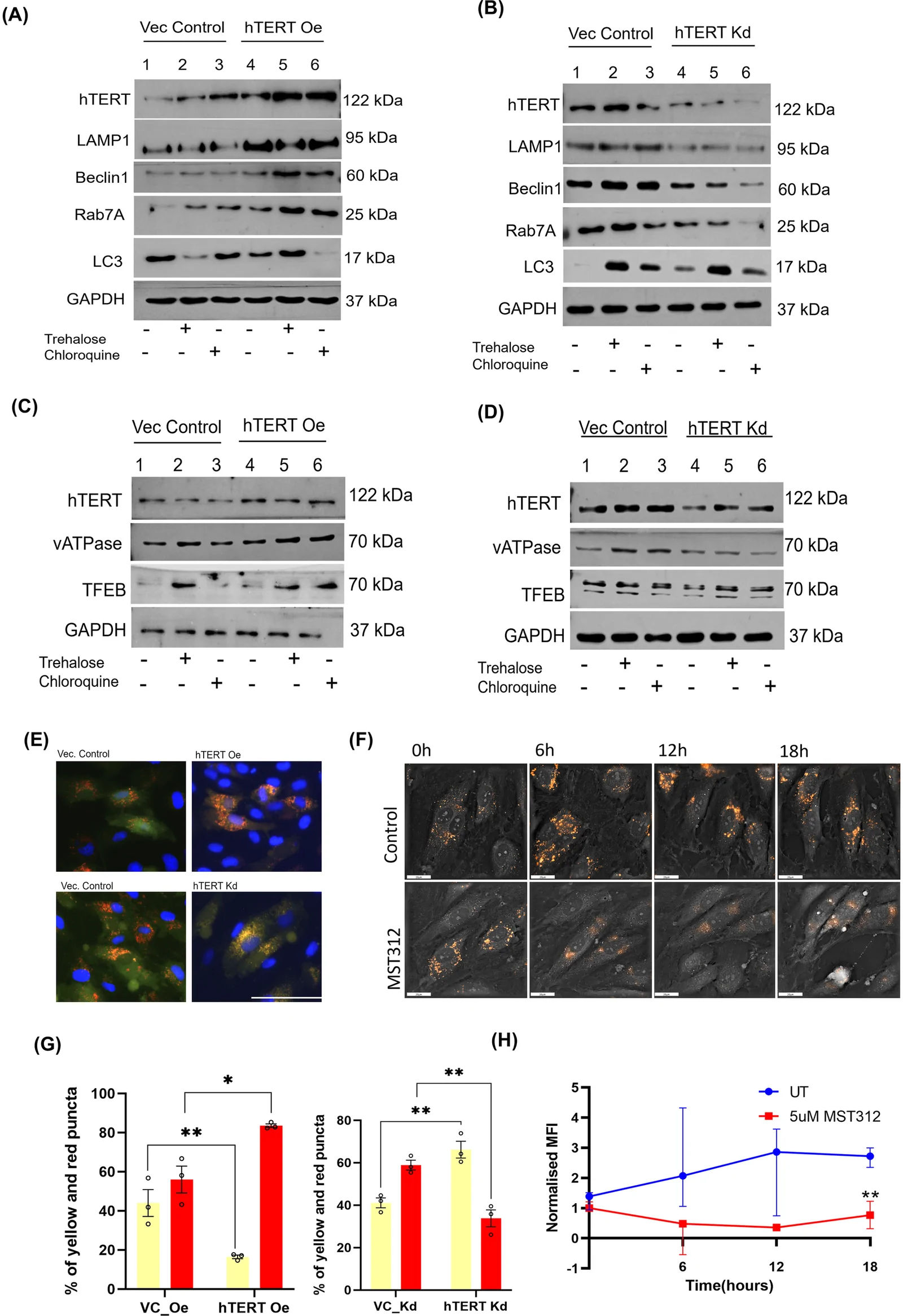
hTERT regulates autophagy kinetics. **(A–D)** Western blots showing expression of autophagy related proteins under 24 h of 100 mM Trehalose (activator) and 5 µM chloroquine (inhibitor) treatment in HUVEC cells with hTERT overexpression (hTERT Oe) and hTERT knockdown (hTERT Kd). Vec control represents respective vector controls used in transfection. Data are representative of three independent replicates. **(E,G)** Fluorescent images of HUVEC cells transfected with mCherry-GFP-LC3 dual reporter plasmid under different conditions and quantification of yellow and red puncta (as indicated by colored bars) to assess autophagy flux. **(F,H)** Live cell imaging of lysosomes in HUVEC cells using Nanolive™ 3D cell explorer and quantifications across different time points. All data are representative of *n* = 3 biological replicates. Unpaired two-tailed t-test **p < 0.005, ****p < 0.00005. Data are presented as Mean ± SEM, Scale bar =75 µm **(E)** Scale bar =20 µm **(F)**.

To understand the lysosomal kinetics, we performed live cell imaging of lysosomes using Nanolive ™ 3D cell explorer. Live cell imaging showed reduction of lysosomes in HUVEC cells as early as 6 h from hTERT activity inhibition using 5 µM MST312 (Figure 4F; Supplementary Videos S1, S2). There was a significant reduction in lysotracker signal by 18 h of live cell imaging (log2FC = −1.83, *p* < 0.005; Figure 4H).

Autophagy is mainly regulated by mTOR pathway. Phospho-mTOR was observed to be reduced upon hTERT Oe as well as under autophagy activation using trehalose indicating that hTERT Oe alone induces autophagy (Supplementary Figure S6C). Since hTERT expression is directly regulated by Akt signaling we looked at Akt phosphorylation upon hTERT Oe. hTERT Oe alone, however, showed reduction in Akt phosphorylation indicating a feedback inhibitory mechanism (Supplementary Figure S6C, Lane 4). Akt is known to directly regulate mTOR and our data showed a simultaneous reduction in both phosphorylation of Akt and mTOR (Supplementary Figure S6D). Densitometric analysis of the protein expression levels are shown in Supplementary Figure S6A,B.

To show that rescue of autophagy processes translates to functionality of cells, we imaged intracellular oxLDL levels under autophagy modulation with hTERT Oe and hTERT Kd cell using DiI labelled oxLDL imaging. hTERT Oe cells showed reduced intracellular oxLDL as compared to vector control (FC = 0.7, *p* < 0.005). Trehalose treatment in hTERT Oe cells had an additive effect on intracellular oxLDL reduction. Chloroquine treatment showed accumulation of oxLDL under control conditions; the accumulation was reduced upon hTERT overexpression (FC = 0.62, *p* < 0.005; Figures 5A,C). hTERT Kd cells showed higher accumulation of oxLDL particles at basal level (FC = 1.44, *p* < 0.005) and a significant accumulation was observed upon chloroquine treatment in hTERT Kd cells (FC = 1.77, *p* < 0.005; Figures 5B,D). Taken together, hTERT Oe demonstrates the cumulative effect of autophagy activation and rescue of endothelial cell dysfunction.

**Figure 5:**
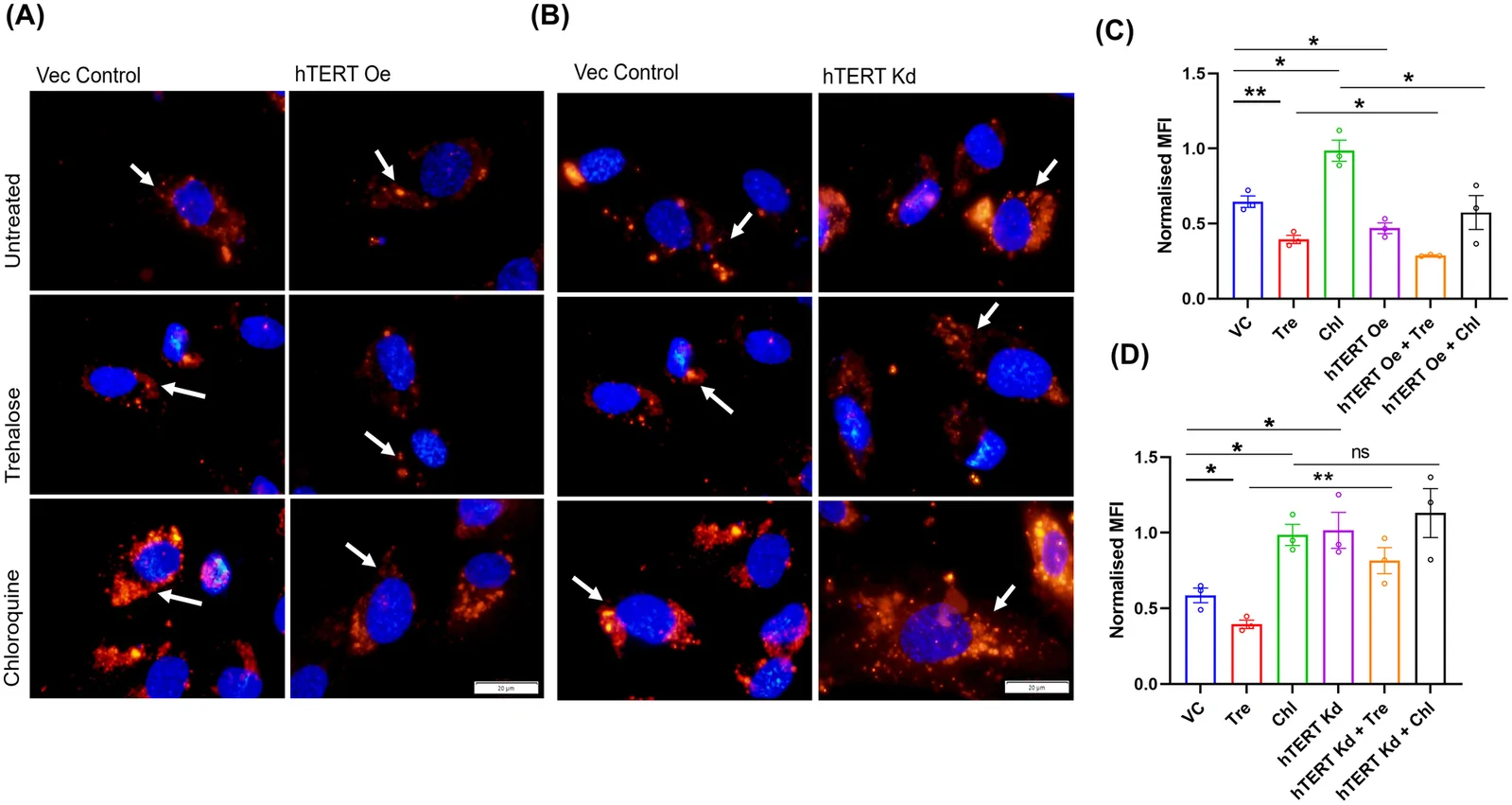
hTERT regulates intracellular oxLDL levels through autophagy. **(A,B)** Images showing DiI labelled oxLDL in HUVEC cells under different conditions. Images were acquired post 1 h of incubation with DiIoxLDL 48 h post transfection. White arrows indicate oxLDL. Scale bar =20 µm **(C,D)** Mean fluorescence intensity (MFI) quantification across biological repeats (Tre = Trehalose, Chl = Chloroquine). Scale ba*r* = 20 µM Data are representative of *n* = 3 biological replicates. Data presented as Mean ± SEM, Unpaired two-tailed t-test, ns = non-significant, *p < 0.05, **p < 0.005.

### 3.6 hTERT associates with Rab7A to regulate autophagy

To investigate the spatial association between hTERT and Rab7A, immunofluorescence colocalization assays were performed in HUVECs under basal and atherogenic stress conditions. A marked increase in colocalization between hTERT and Rab7A was observed following 96 h of oxLDL treatment compared to untreated controls, suggesting recruitment of hTERT to late endosomal compartments (Figures 6A,B). Quantification of colocalization using Pearson's correlation via ImageJ analysis supported this observation, indicating a statistically significant enhancement of hTERT-Rab7A interaction upon oxLDL exposure (FC = 1.42, *p* ≤ 0.05) (Figure 6C). To elucidate hTERT's interaction network in autophagy regulation, co-immunoprecipitation (Co-IP) assays were performed under basal conditions. Co-immunoprecipitation assays revealed that hTERT specifically associated with Rab7A and Beclin1, but not with the lysosomal marker LAMP1 (Figure 6D). Strikingly, oxLDL treatment for 96 h led to an increase in hTERT binding to Rab7A (Figure 6E), supporting the hypothesis that hTERT is recruited to autophagy regulatory complexes in response to atherogenic stimuli. Since LAMP1 did not bind to hTERT, we used LAMP1 as a loading control for Co-IP experiments. To assess the dependency of hTERT and Rab7 in regulating autophagy, we transfected HUVEC cells with dominant negative Rab7 (Rab7DN) along with hTERT modulation to study intracellular oxLDL levels, Rab7DN transfected cells showed higher intracellular levels as compared to controls indicating deficient autophagy. Overexpressing hTERT along with Rab7DN did not rescue the oxLDL levels, showing interdependency of hTERT and Rab7A in regulating autophagy. hTERT knockdown coupled with Rab7DN showed highest accumulation of intracellular oxLDL (Figures 6G,H). This interaction pattern suggests hTERT's preferential involvement with early-to-late autophagy machinery rather than terminal lysosomal compartments (Figure 6I).

**Figure 6:**
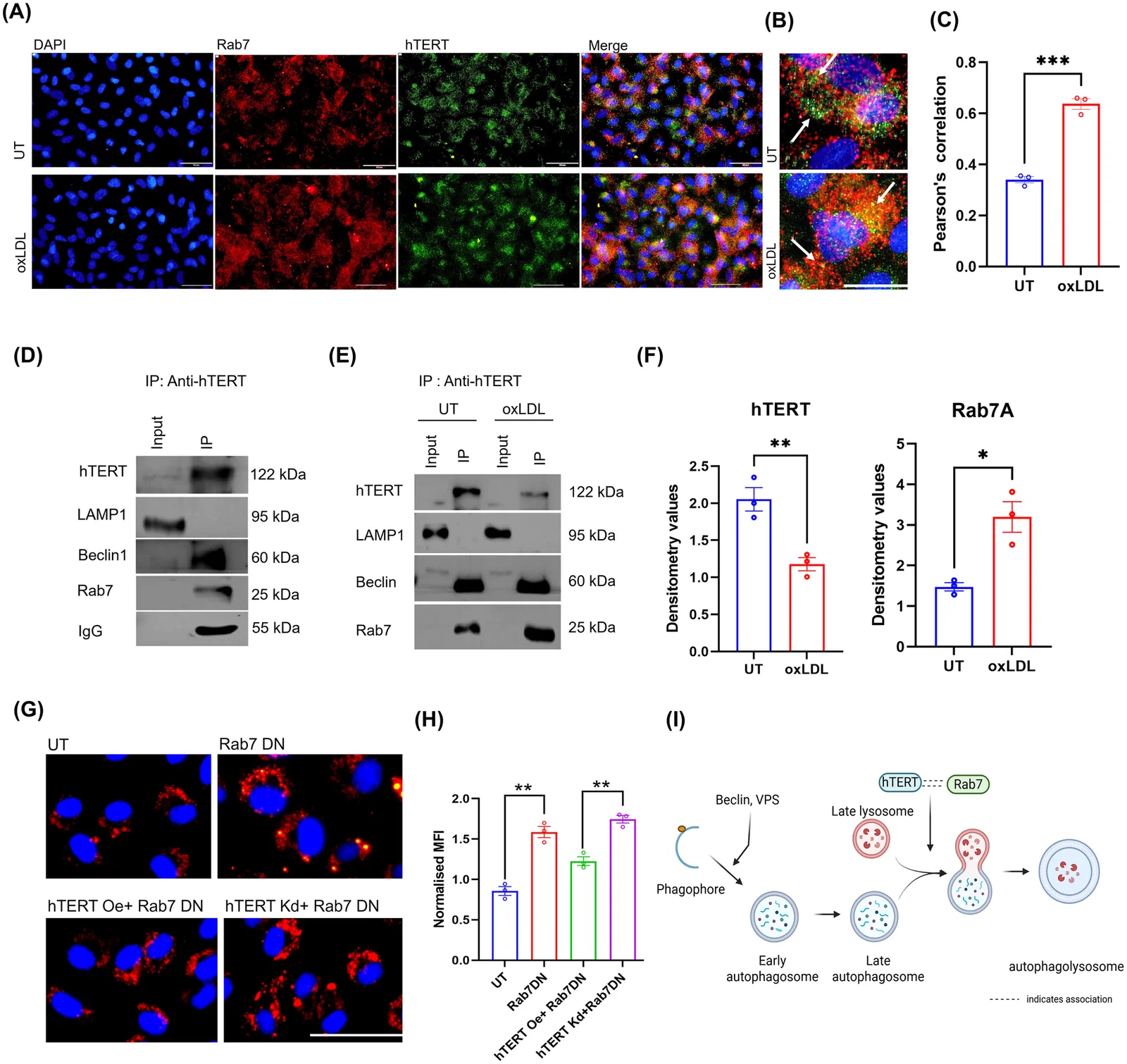
hTERT associates with Rab7A to regulate autophagy. **(A)** Immunofluorescence images showing expression of hTERT and Rab7A under 40 µg/mL oxLDL treatment of HUVEC cells **(B)** Enlarged images showing colocalization of hTERT and Rab7A upon oxLDL treatment indicated by arrows. **(C)** Graph indicates quantification of colocalization using Pearson's correlation plugin in Fiji software. **(D,E)** Co-immunoprecipitation experiments showing association of hTERT with autophagy related proteins at basal **(D)** oxLDL treated condition **(E,F)** Densitometric analysis of hTERT and Rab7A shown in western blots in **(E) (G)** Enlarged images of DiI-oxLDL in HUVEC cells under Rab7DN, hTERT overexpression (hTERT Oe) and hTERT knockdown (hTERT Kd) conditions. **(H)** Quantification of DiI-oxLDL fluorescence intensity under different conditions **(I)** Schema showing possible association step of hTERT in autophagy regulation (Created using BioRender.com). Scale bar=100 µm **(A)**, 50 µm **(B,F)**, Data are representative of *n* = 3 biological replicates, Data are presented as Mean ± SEM, Unpaired two-tailed t-test, *p < 0.05, **p < 0.005, ***p < 0.0005.

## 4 Discussion

In the current study, we investigated whether human telomerase reverse transcriptase (hTERT) may improve endothelial function, in part through regulation of autophagy. Chronic oxLDL exposure significantly reduced hTERT expression in endothelial cells without significant alterations in relative telomere lengths implicating a non-canonical role of hTERT in the endothelial damage process. Pharmacological inhibition of hTERT using MST312 mimicked these effects, indicating that loss of hTERT function can partially induce endothelial dysfunction. Furthermore, combined treatment with oxLDL and hTERT inhibition synergistically exacerbated endothelial dysfunction, as evidenced by elevated ICAM1 expression. Conversely, overexpression of hTERT in HUVECs conferred a protective effect by restoring endothelial function, lowering intracellular oxLDL levels and reducing ICAM1 levels. Functional assays such as monocyte attachment assay, TEER and barrier breach assays further confirmed that hTERT overexpression ameliorates oxLDL-induced endothelial barrier dysfunction, providing a mechanistic link with atherosclerosis. Although MST312 used is most specific to TERT, it can have minimal off target effects independent of its main role. However, the effects observed on endothelial cells in this study might not be associated with the reported off target effects of MST312 such as suppression of SHH signaling or lower expression of Lamin A (35, 36).

The presence of senescent endothelial cells has been well established in the coronary arteries of patients with ischemic heart disease, demonstrated by the presence of *β*-galactosidase-positive cells in arterial beds. This direct assessment draws a clear association between endothelial senescence, endothelial dysfunction, and the pathogenesis of vascular diseases (37, 38). Additionally, patients with coronary artery disease exhibit both reduced telomere length and lower T/C ratios, implicating telomerase insufficiency as a causal factor in vascular pathology (39). While these studies investigate the canonical functions of hTERT in vascular disease, our data highlights additional non canonical mechanisms through which hTERT maintains vascular homeostasis.

Autophagy has been implicated in the pathogenesis of atherosclerosis, and strategies aimed at restoring autophagic flux, through overexpression of transcription factor TFEB as well as enhancing it through trehalose treatment have been shown to alleviate atherosclerosis (13, 15). However, the studies do not offer mechanistic insights into the causal factors pertaining to dysregulated autophagy. In glioblastoma, hTERT modulates basal autophagy, through regulation of Beclin 1 (29). More recently, interaction between telomerase and autophagy pathway has been shown to mediate flow mediated arteriolar vasodilation, suggesting that autophagy may be acting downstream of Telomerase (28). Our results extend these findings to primary endothelial cells and also delineate the underlying mechanisms, with hTERT modulating the expression and dynamics of key autophagy-related proteins, including LAMP1, Rab7A, v-ATPase, and transcription factor EB (TFEB). Live-cell imaging of lysosomes revealed a reduction in lysosomal content in MST312-treated cells, suggesting an impaired autophagic flux upon hTERT inhibition. In contrast, trehalose-treated, hTERT-overexpressing HUVECs retained the lowest levels of intracellular oxLDL, whereas hTERT-deficient cells accumulated oxLDL at levels comparable to those observed in chloroquine-treated cells. The combination of hTERT knockdown and chloroquine treatment resulted in maximal oxLDL accumulation, indicating that hTERT is a critical regulator of autophagy-mediated clearance.

Investigating this function revealed a novel association between hTERT and Rab7A as well as Beclin 1 under basal and oxLDL-stimulated conditions. Given that hTERT contains a BH3-like domain, its interaction with Beclin 1 is consistent with known interactions between Bcl-2 family proteins and autophagy regulators (40, 41). While an association between hTERT and Rab7A has not been previously described, our findings raise the possibility that hTERT, Rab7A, and Beclin 1 form a regulatory complex that governs late-stage autophagy and lysosomal trafficking in endothelial cells. The observed hTERT–Rab7A association therefore supports a model of stress-responsive autophagy regulation in HUVECs, but confirmation in arterial endothelial cells or human vascular tissue will be required.

hTERT might serve a dual role by maintaining basal autophagy and facilitating autophagy associated mechanisms for oxLDL clearance during cellular stress. Thus, the age-associated reduction in hTERT levels might be the molecular link between inefficient autophagy and endothelial dysfunction in individuals of advanced age who are susceptible to atherosclerosis. However, further experiments using aged arterial endothelium and targeted analyses of human arterial tissue would validate our mechanistic findings. The study thus expands, the functional repertoire of hTERT beyond telomere maintenance in maintaining endothelial homeostasis and identifies hTERT-Rab7A axis as a candidate mechanism to promote protection against endothelial dysfunction under atherogenic stress.

## Data Availability

The original contributions presented in the study are included in the article/Supplementary Material, further inquiries can be directed to the corresponding author.
